# Reevaluation of the Prevalence of Metabolic Syndrome in an Urban Area of Turkey

**DOI:** 10.4274/Jcrpe.778

**Published:** 2013-03-21

**Authors:** Mehmet Emre Atabek, Beray Selver Eklioğlu, Nesibe Akyürek

**Affiliations:** 1 Necmettin Erbakan University, School of Medicine, Department of Pediatric Endocrinology, Konya, Turkey

**Keywords:** Childhood obesity, metabolic syndrome, insulin resistance, impaired glucose tolerance, type 2 diabetes

## Abstract

**Objective:** Our aim was to reveal a change in the prevalence of metabolic syndrome (MS) in the province of Konya in five years.

**Methods:** We studied 202 obese children and adolescents (body mass index >95th percentile) aged between 7 and 18 years. The diagnosis of impaired glucose tolerance, type 2 diabetes mellitus (T2DM), and MS were defined according to the modified World Health Organization criteria adapted for children.

**Results:** MS was found in 56.4 % with a significantly higher rate among adolescents aged 12-18 years (63.2%) than among prepubertal children aged 7-11 years (47%) (p=0.01). The prevalence figures for insulin resistance, glucose intolerance, and T2DM were 60, 8, and 2% among prepubertal children and 81.8, 12.8, and 0% among adolescents, respectively. The prevalence of fasting hyperinsulinemia in adolescents was significantly higher than in prepubertal children (p<0.001). Hypertension was significantly more common in adolescents (42.8%) than in prepubertal children (32.9%) (p=0.04).

**Conclusions:** We found that the incidence of MS in the city center of Konya approximately doubled in the last five years with increased rates of morbidity and abnormal lipid profiles.

**Conflict of interest:**None declared.

## INTRODUCTION

Obesity is a chronic condition which impairs the quality of life in many ways. The prevalence of obesity in childhood today is more than 10 times compared to the 1970s. The World Health Organization (WHO) reported that overweight and obesity are responsible for 80%, 35%, and 55% of cases of type 2 diabetes mellitus (T2DM), ischemic heart disease, and hypertension, respectively (1). Obesity is especially associated with insulin resistance, hyperinsulinemia, T2DM, and cardiovascular diseases (2).

Metabolic syndrome (MS) is one of the most important complications of obesity. MS is defined as a clinical condition intertwined with T2DM, cardiovascular disease, hypertension, dyslipidemia, and insulin resistance (3). Genetic and environmental factors also have a role in its development. MS increases the risk of T2DM fivefold and the risk of coronary heart disease twofold (4).

MS prevalence varies according to diagnostic criteria and populations. According to the NHANES III survey covering the period of 1988-1994, the prevalence of MS was 28.7% in obese adolescents in the United States of America (5). In Turkey, MS prevalence was reported to vary between 2.2 and 20% in childhood (6,7). In a previous study (8), we found the MS prevalence as 27.2% in children and adolescents.

Our aim in this study was to determine the prevalence of MS in the urban area of Konya and to compare the results with previous findings.

## METHODS

Two hundred and two children (105 females and 97 males, aged 11.65±3.11 years), who presented to the outpatient clinic of the Department of Pediatric Endocrinology and Diabetes at Necmettin Erbakan University Research Center in Konya, Turkey with the complaint of obesity, were included in our study. The children were required to meet the following inclusion criteria: (1) age, 7-18 years; (2) body mass index (BMI), greater than the 95th percentile for age and gender, according to the standards of the Centers for Disease Control and Prevention; (3) absence of a prior major illness, including type 1 or 2 diabetes, or of a condition known to influence body composition, insulin action, or insulin secretion; 4) absence of a history of medication known to influence metabolism such as glucocorticoid therapy. Informed consent and assent were obtained from all parents and children, respectively.

Each child underwent a complete physical examination, including anthropometric measures. Their pubertal development stages were assessed by a single pediatric endocrinologist using the criteria of Tanner. Height and weight were measured with an empty bladder in post-absorptive conditions. Height was measured to the nearest 0.5 cm on a standard height board. Weight was determined to the nearest 0.1 kg on a standard physician’s beam scale with the subject dressed only in light underwear and no shoes. BMI was calculated as weight (in kilograms) divided by height (in meters) squared. Waist circumference was measured at the level of the umbilicus with the patient in the standing position and breathing normally. Hip circumference was measured at the level of the iliac crest. Blood pressure was measured with a standard mercury sphygmomanometer after the subjects had rested for at least 10 min.

An oral glucose tolerance test (OGTT) was performed in 128 of these obese children and adolescents using a dose of 1.75 g/kg body weight (to a maximum of 75 g). The initial venous blood samples were obtained in the morning by venipuncture after overnight fasting. Subsequent samples were taken at 0, 30, 60, 90, and 120 minutes after glucose loading. Plasma glucose and plasma insulin levels were determined in these samples: plasma glucose by the glucose oxidase method and plasma insulin using IMMULITE immunoassay (IMMULITE Diagnostic Products Corporation, Los Angeles, CA). Plasma concentrations of total cholesterol, triglycerides, low-density lipoprotein (LDL)-cholesterol and high-density lipoprotein (HDL)-cholesterol were also measured in the initial samples using routine enzymatic methods with an Olympus 2700 Analyzer (Olympus Diagnostica GmbH, Ireland).

Criteria for abnormal glucose homeostasis were defined according to the modified WHO criteria adapted for children (9). The corresponding categories, when the OGTT is used, are the following: normal glucose tolerance is defined as a 2-hour post-load glucose (2 hour PG) level of <140 mg/dL (<7.8 mmol/L); impaired glucose tolerance (IGT) as a 2 hour PG level between 140 mg/dL (7.8 mmol/L) and 200 mg/dL (<11.1 mmol/ L), and a diabetic state as a 2 hour PG ≥200 mg/dL (≥11.1 mmol/L). Following American Diabetes Association recommendations, a fasting glucose ≥110 mg/dL is defined as imparied fasting glucose (IFG) and ≥126 mg/dL (≥7.0 mmol/L) as diabetes.

Homeostasis model assessment of insulin resistance (HOMA-IR; fasting insulin x fasting glucose/22.5), which correlate with estimates of insulin resistance measured by the euglycemic clamp technique, was used as an index of insulin resistance (10). Insulin resistance is defined as HOMA-IR of greater than 3.16 according to our previously published data (11). Hyperinsulinism was defined from norms for pubertal stage: prepubertal >15 mU/L and midpuberty (stages 2-4) >30 mU/L (12). Fasting glucose/insulin ratio was calculated as fasting glucose concentration (mg/dL) /fasting insulin concentration (μU/mL). Quantitative insulin sensitivity check index was calculated as 1/ [(log fasting insulin concentration (μU/mL) + log fasting glucose concentration (mg/dL)] (13).

MS was defined according to the WHO criteria adapted for children, a definition which requires three or more of the following components (14).

(1) Obesity: BMI >95th percentile for age and sex.

(2) Abnormal glucose homeostasis: Any of the following (a) fasting hyperinsulinemia; (b) IFG; (c) IGT.

(3) Hypertension: Systolic blood pressure >95th percentile for age and sex.

(4) Dyslipidemia: Any of the following (a) high triglycerides (>105 mg/dL (>1.2 mmol/L) in children <10 years of age, and >136 mg/dL (>1.5 mmol/L) in children ≥10 years of age); (b) low HDL-cholesterol (<35 mg/dL (<0.9 mmol/L)); ?(c) high total cholesterol (>95th percentile).

**Statistical Analysis**

Data were expressed as mean±standard deviation. IGT, insulin resistance, T2DM and MS prevalence rates according to the prepubertal and pubertal groups were estimated by chi-square and Fisher tests. The differences between data were estimated using the student’s t-test. Statistical significance was taken as p<0.05. All statistical analyses were performed using the Statistical Package for Social Sciences (SPSS/Windows Version 11.0, SPSS Inc. Chicago, IL, USA).

## RESULTS

A total of 202 children and adolescents aged 7-18 years (85 prepubertal and 117 pubertal) underwent assessment. Mean age was 11.65±3.11 years. The clinical characteristics of the study population are given in Table 1.

According to our results from OGTT performed in 128 obese children and adolescents, insulin resistance (HOMA-IR >3.16) was observed in 72.2%, IFG in 4.4%, and IGT in 10.9%.

MS, defined as presence of ≥3 components, was found in 114 subjects (56.4%), with a significantly higher rate among pubertal subjects (63.2%) than among prepubertal subjects (47%) (p=0.01). There were no significant differences in the prevalence of MS by gender. Table 2 shows the prevalence of individual components of cardiovascular risk factors and MS by pubertal status.

**Figure 1:**
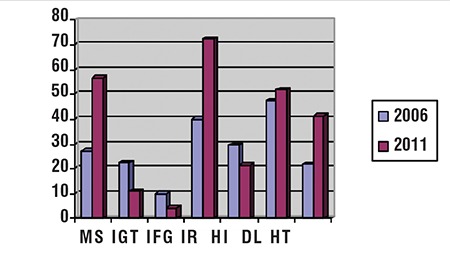
Prevalence of MS and of its components in obese children and adolescents

In the present study, the prevalences of IR, IFG and IGT were 60%, 5.8% and 8% in the prepubertal age group and 81.1%, 3.4% and 12.8% in the pubertal age group, respectively. The differences in the rates of IR between prepubertal and pubertal children were significant, whereas the differences in IFG and IGT were not significant (p<0.001, =0.41 and =0.56, respectively). The prevalence of T2DM was 2% among the prepubertal group. Hyperinsulinemia was also more frequent in pubertal children (27.3% vs. 14.1%; p<0.001). Hypertension was significantly more common in adolescents (47.8%) than in prepubertal children (32.9%) with obesity (p=0.04). Overall dyslipidemia was identified in prepubertal and pubertal groups as 52.9% and 51.2%, respectively, with no significant differences (p=0.86). High total serum cholesterol concentrations were noted in 92.8%, high serum triglyceride concentrations in 29.4% and low HDL in 23.1% of the prepubertal subjects. In the pubertal age group, high total serum cholesterol concentration was found in 92.9%, high serum triglyceride concentration in 30.4% and low HDL in 26.5% of the subjects. Comparison of previous and current data was shown in Figure 1.

## DISCUSSION

It is known that obesity is a global problem and leads to increased morbidity and mortality. Many studies report an increased prevalence of glucose abnormalities and especially MS in obese children. Different prevalence figures for glucose abnormalities and MS in obese children were reported from Turkey (8). In this present study, we aimed to reveal a change in the prevalence of MS in the province of Konya in the past five years.

Although the prevalence figures differ by diagnostic criteria, the overall prevalence of MS in children is estimated to be 3-4% (15). According to Cizmecioglu et al (16), 38.8% of obese children in Turkey were diagnosed as having MS. Isomaa et al (17) found this prevalence to be 42% (17). According to the results of Cook et al (5), the prevalence of the MS in adolescents was 28.7%. The gross prevalence of the MS in the USA obese population was 30% after age-adjustment (18). We found 47% (40) of obese children and 63.2% (74) of adolescents, 56.4% (114) of all patients to have evidence of MS as defined above by the presence of three of the following components: obesity, abnormal glucose homeostasis, dyslipidemia and hypertension.

Five years ago, we found MS prevalence as 27.2 in obese prepubertal children and 37.6% in obese adolescents (8). According to our data, we found that the prevalence of MS had increased both in the prepubertal and pubertal group, and the prevalence had doubled in five years. We attribute this increase to changes in lifestyle and nutrition behaviors and spending more time in front of television and computer screens.

The development of T2DM requires both IR and inadequate insulin secretion, leading to persistent hyperglycemia. IR in the young has been reported in a variety of ethnic groups and is strongly associated with obesity (19,20). The stress of obesity and the increased demand for insulin at the time of adolescence explain the largely pubertal and postpubertal onset of type 2 diabetes in children. Insulin sensitivity decreases by 30% during puberty with compensatory increase in insulin secretion (21). In our study, the rate of differences of IR in the prepubertal and pubertal stages was significant and more prevalent among obese adolescents. IR was increased in both prepubertal and pubertal groups as compared to our previous data (9). All insulin sensitivity indices were significantly high in the pubertal group.

Glucose metabolism disorders secondary to obesity are initiated in childhood. In a study from Spain, the prevalence of IGT in obese children and adolescents was 7.4%. There were no children with T2DM; however, the prevalence of IR in obese children studied was 35.8% (22). Wabitsch et al (23) determined the prevalence of T2DM in 1.5% of the patients and impaired glucose regulation in 2.1% of the patients in a large group of Caucasian children and adolescents with obesity. Wiegand et al (24) found that 2.4% of their obese pediatric patients had IGT. In an Italian study, the prevalence rates of T2DM and IGT were 0.1 and 4.5%, respectively (25). In our study, we found that 14 (10.9%) patients had IGT and 1 (0.7%) patient was diagnosed with T2DM. It is reported that the prevalence of IGT is not influenced by obesity but related to poor beta-cell function that shows signs of deterioration with age (26). The evolution from normal to IGT is associated with IR and a failure of beta-cell insulin secretory capacity, which deteriorates further as T2DM develops (27). Our findings of a high prevalence of IGT and IR but a low prevalence of T2DM reflect the range of abnormalities of glucose homeostasis associated with obesity in childhood.

Elevated triglycerides and low levels of HDL-cholesterol characterize the dyslipidemia in MS. Increased triglycerides in the presence of IR and hyperinsulinemia result from increased circulating free fatty acids. As IR increases, the lipolysis inhibitory mechanisms of insulin on adipose tissue diminish and more free fatty acids are produced (28). In our study, total cholesterol and triglyceride serum concentrations were higher and HDL level was lower in both obese children and adolescents; however, the differences were not statistically significant. Dyslipidemia frequency was 51.9%, which is higher according to our previous data and studies performed by Saha et al and Viner et al (29,30).

The cause of hypertension in the MS is multifactorial and likely to be related to all the elements of the syndrome including obesity, IR, and dyslipidemia. Obesity may be the most important factor, but the other elements of the syndrome also play a role in creating and mediating the changes that ultimately result in hypertension (31). In our study, 41.5 % of all patients had hypertension. Sorof et al (32) found the hypertension prevalence as 10.7% in obese children. However, increased figures (47-60%) were reported for hypertension prevalence with ambulatory blood pressure monitoring (33). Although we took one measurement of blood pressure, we observed a high frequency. These findings show that preventing obesity is one of the important factors for the development of hypertension and related end-organ damage in early ages.

The prevalence of IR is very high among obese children, but T2DM is yet to develop in this age group. In conclusion, the incidence of MS in the city center of Konya province approximately doubled over the last five years, and we found increased rates of morbidity.

## Figures and Tables

**Table 1:**
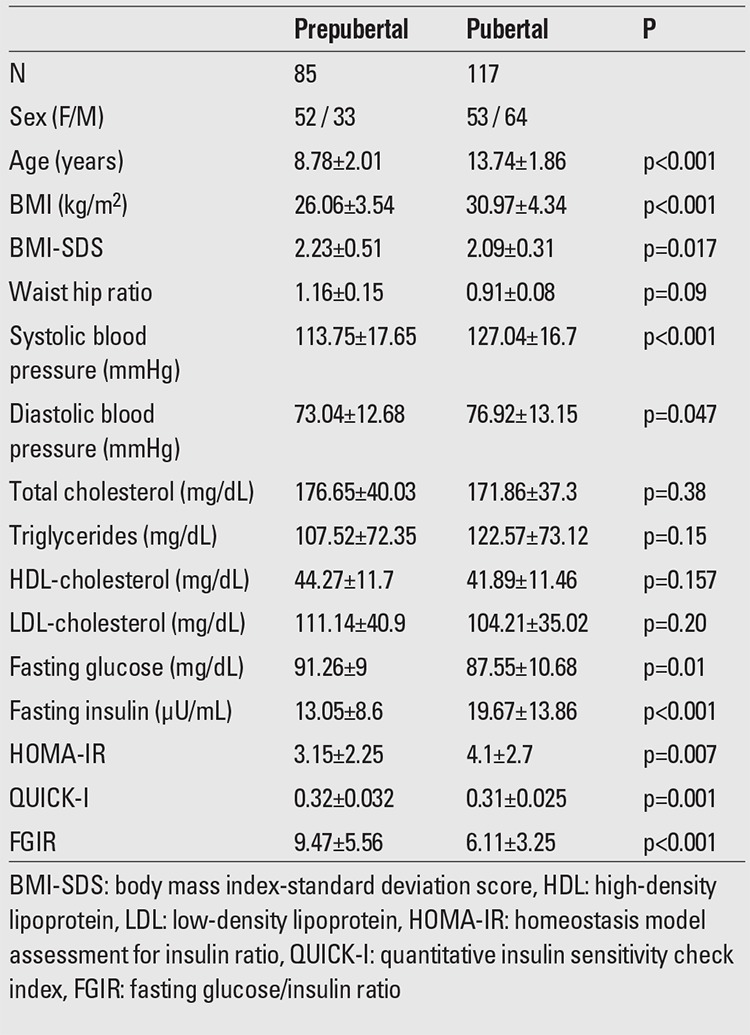
Clinical characteristics of the study population

**Table 2:**
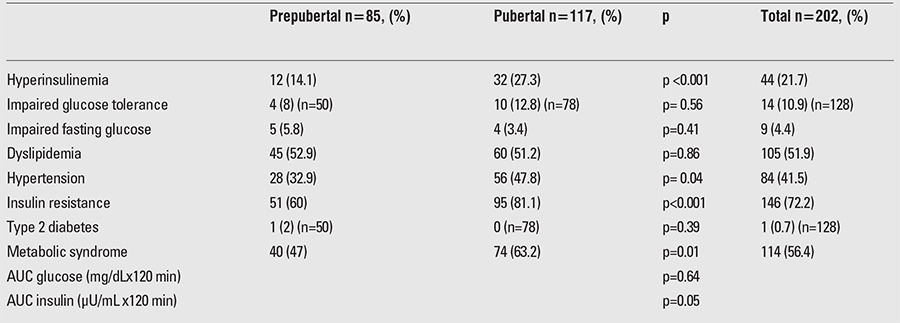
Prevalence of risk factors and metabolic syndrome by pubertal status
